# Silver ion-induced fluorescent quenching in aqueous graphene oxide suspensions

**DOI:** 10.1039/d6na00039h

**Published:** 2026-08-03

**Authors:** H. M. C. S. Wijerathne, Nadeeka D. Tissera, G. Priyadarshana, D. Dahanayake, K. M. Nalin de Silva, Ruchira N. Wijesena

**Affiliations:** a Department of Textile and Clothing Engineering, Institute of Technology, University of Moratuwa Diyagama Homagama 10200 Sri Lanka ruchiraw@itum.mrt.ac.lk; b Department of Chemistry, Faculty of Science, University of Colombo Colombo 00700 Sri Lanka; c Department of Materials and Mechanical Technology, Faculty of Technology, University of Sri Jayewardenepura Pitipana Homagama 10200 Sri Lanka; d Faculty of Science, NSBM Green University Diyagama Homagama 10200 Sri Lanka

## Abstract

In this work, we demonstrate that the fluorescence of aqueous graphene oxide (GO) suspensions is significantly quenched upon the introduction of silver ions (Ag^+^). The extent of quenching exhibits a clear dependence on both Ag^+^ concentration and interaction time. Comprehensive characterization using TEM, SEM, AFM, and UV-visible spectroscopy confirms that Ag^+^ ions undergo *in situ* reduction and subsequently deposit onto the GO surface. Altogether, GO was shown to drive the spontaneous reduction of silver ions to silver nanoparticles while providing a support structure for the particles to nucleate and grow. The reduction mechanism was attributed to galvanic displacement driven by the relative reduction potential difference between GO and silver. An ultraviolet photoelectron spectroscopy study was carried out on GO to support this hypothesis. Finally, the type of interaction between silver and GO was investigated using FT-IR and XPS studies. Both studies confirmed that the main point of interaction is through the hydroxyl moieties of GO. Based on the obtained data, three possible mechanisms for fluorescent quenching behaviour were presented. It's anticipated that GO would provide a convenient pathway for silver ion sensing and silver-based nanophotonic applications through the discussed interaction pathways.

## Abstract 2

In this work, we demonstrate that the fluorescence of aqueous graphene oxide (GO) suspensions is significantly quenched upon the introduction of silver ions (Ag^+^).

## Graphical abstract

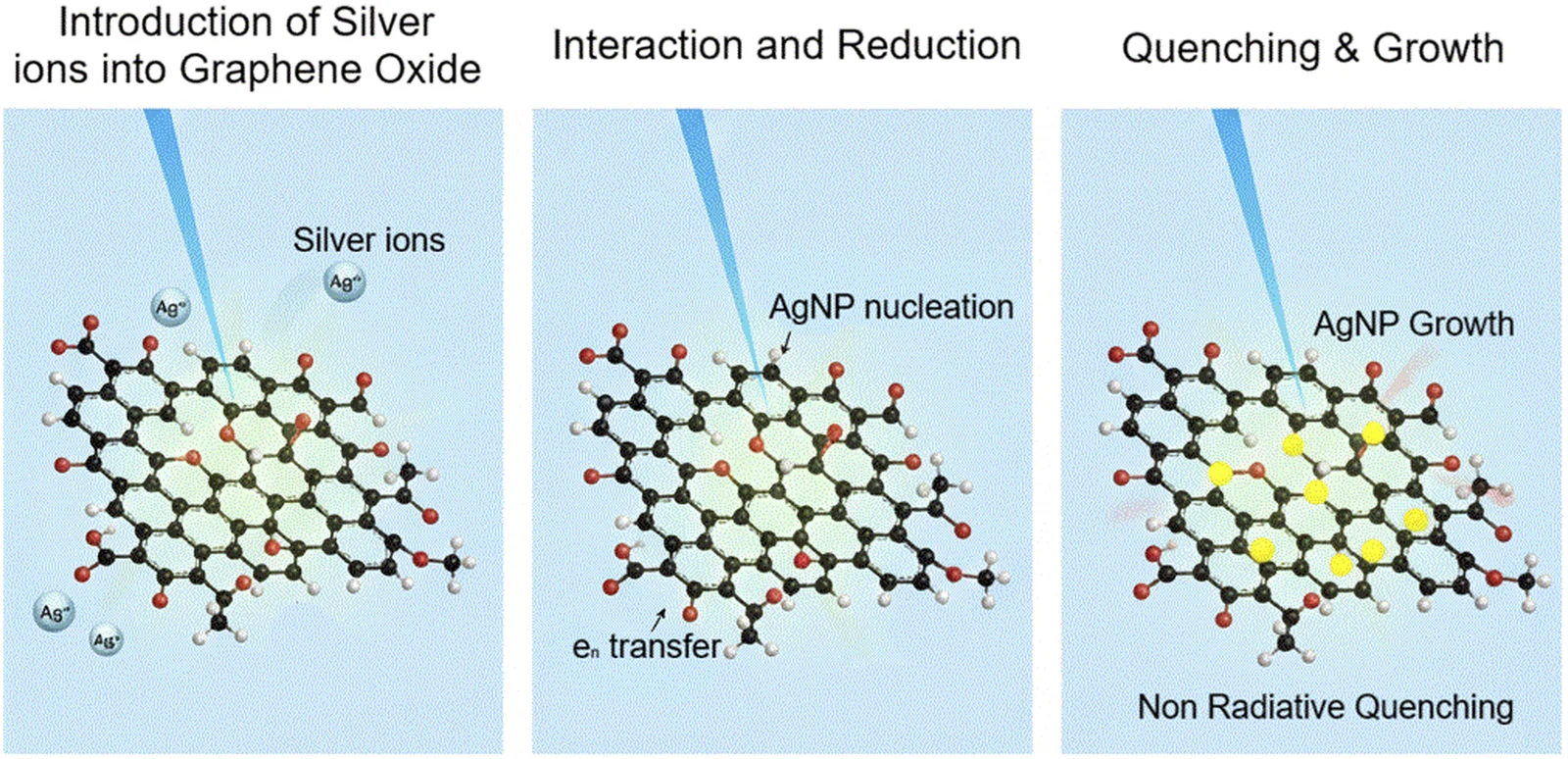

## Introduction

Since its first isolation in 2004, graphene has emerged as a major focus of both fundamental and applied research owing to its exceptional electronic, mechanical, and thermal properties.^1–4^ Intensive research efforts worldwide have focused on integrating graphene and related two-dimensional (2D) materials into real-world applications, including electronics, composites, energy storage and conversion, drug delivery, and catalysis.^4–12^ Graphene oxide (GO) is a well-known precursor material for graphene, consisting of graphene layers modified with oxygenated functional groups on their basal planes and edges.^13–17^ It's well known that these oxygen-bearing functional groups can interact with other organic or inorganic species through covalent, non-covalent, or ionic interactions.^18–22^ Among these, noble metal interactions with the GO surface are of special importance due to their potential use in catalysts, fuel cells, biosensors, spectroscopic applications, and transparent conductors.^23–25^

Although graphene is intrinsically a zero-band gap semimetal, visible and near-infrared fluorescence of aqueous exfoliated GO suspensions and cast sheets was reported by a few groups.^26–32^ Unlike pure graphene, where all its carbon atoms are sp^2^ hybridized, GO contains sp^2^- and sp^3^-hybridized carbon atoms covalently bonded to oxygen-bearing functional groups. These sp^3^-hybridized regions are known to surround small sp^2^-hybridized clusters. Radiative recombination between photoexcited e–h pairs is known to be the reason for the photoluminescence (PL) behaviour of GO.^30,32–34^ In another view, it's suggested that GO PL can also be attributed to a number of molecular fluorophores that may consist of carboxylic acid groups and nearby localized graphene regions.^27^ This conclusion was primarily drawn from the fact that the PL behaviour of GO is strongly pH dependent. It's generally accepted that both these phenomena will contribute to the PL behaviour. Photoluminescence phenomena of GO can be exploited to open up several different photonics applications. Unlike carbon nanoribbons and carbon quantum dots, which are relatively hard to fabricate and modify the bandgap, GO can be easily synthesized by using low-cost wet chemical routes. To date, the majority of work exploiting the GO photoluminescence behaviour has been concentrated around imaging.^26,35^

Many studies utilizing the fluorescence behaviour of GO have been reported, mainly in the areas of imaging,^26,28^ biosensing,^36–38^ ion detection,^39–41^*etc.*

In this paper, we explore the effect of Ag^+^ ions on the PL behaviour of GO. Here, we report that the introduction of Ag^+^ ions into exfoliated GO dispersions significantly reduces PL intensity. An explanation was brought forward to explain this behaviour with supporting experimental data.

## Materials and methods

### Materials

All the chemicals used in the experiments were reagent grade and purchased from Sigma-Aldrich. The graphite used in the experiment was a kind gift from ASBURY CARBONS.

### Preparation of GO

The preparation of GO was carried out by using the improved Hummers' method.^42^ A 9 : 1 (v/v) mixture of concentrated H_2_SO_4_/H_3_PO_4_ (360 mL : 40 mL) was added to a mixture containing graphite flakes (3.0 g) and KMnO_4_ (18.0 g). The slight exothermic increase in temperature (35–40 °C) was controlled using a water bath. The reaction mixture was then heated to 50 °C and stirred for 12 h, after which it was allowed to cool to room temperature. Subsequently, the reaction mixture was poured onto ice (400 mL) containing 30% H_2_O_2_ (3 mL). The resulting dispersion was centrifuged at 2000 rpm for 30 min, and the supernatant was discarded. The remaining solid was sequentially washed with 200 mL of water, 200 mL of 30% HCl, and 200 mL of ethanol. After each washing step, the mixture was centrifuged at 2000 rpm for 30 min. When the successive washing was completed, the GO suspension was centrifuged one more time to prepare a thick slurry. The solid content of the slurry was determined by drying a known weight until a constant weight was reached. This GO slurry was used as the GO source in all the experiments with the indicated dilutions.

### Preparation of GO-Ag mixed solutions

GO dispersions were prepared in distilled water at a concentration of 50 µg mL^−1^. Accurately weighed GO was first sonicated in an ultrasonic bath for 30 min and subsequently stirred for 2 h. After sonication, no particulate matter was visually observed. Before preparing any mixed solutions, each dispersion was briefly sonicated to ensure uniformity. The as-prepared GO dispersions exhibited a pH in the range of 4.9–5.5, attributed to residual acidic species remaining from the synthesis process. When required, the pH was adjusted using dilute HNO_3_ or KOH. For AgNO_3_ incorporation, the GO dispersion was divided into 10 mL aliquots, followed by the slow addition of 0.1 M AgNO_3_ solution using a 10 µL micropipette. After mixing, each sample underwent brief sonication for 30 s. For the photoluminescence (PL) and UV-vis quenching experiments, the Ag^+^ concentration was maintained in the range of 0.5–0.8 µM to allow for the precise monitoring of the quenching kinetics and to prevent instantaneous signal saturation.

For microscopy (SEM, TEM, and AFM) and XRD, a concentration of 0.8 mM Ag^+^ was used to ensure adequate nanoparticle density for surface analysis.

All reactions were conducted at 25 °C.

### Material characterization

Fluorescence measurements were obtained using a Horiba Fluorolog FL-1000 fluorometer with 5 nm slit widths for both excitation and emission and 1 nm increments. Integration time for all the measurements was 1 s per point, and the measurements were corrected for detector response and excitation power. Absorbance spectra were recorded using a Shimadzu UV-VIS-NIR UV-3600 spectrophotometer using quartz cuvettes for the spectral range of 190 nm to 800 nm.

Transmission electron microscopy (TEM) was used to characterize the morphology of the prepared samples using a JEOL JEM-2100 operating at an accelerating voltage of 200 kV. Water dispersions of the nanomaterials were cast on TEM grids and allowed to dry. The dried grids were taken for inspection. The surface morphology was analysed using scanning electron microscopy (SEM) using a Hitachi SU6600 Analytical Variable Pressure FE-SEM.

X-ray photoelectron spectroscopy (UPS) and UV photoelectron spectroscopy (UPS) measurements were carried out with an EXCALABXi + hemispherical electron analyser (Thermo Fisher Scientific, United States) using monochromatic Al kα radiation (*hν* = 1486.6 eV), and Thermo Advantage (5.982) software was used to perform curve fitting and to calculate other XPS parameters. Before the analysis, GO dispersions were drop-cast on a glass substrate and allowed to dry.

The UPS spectra were obtained using HeI irradiation with *hν* = 21.22 eV produced by a UV source. During UPS measurements, the analyser worked in the constant retarding ratio (CRR) mode, with CRR = 10. A bias of −10.0 V was applied to the sample to avoid interference from the spectrometer threshold in the UPS spectra.

Zeta potential measurements were also conducted using a Zetasizer Nano ZS (Malvern Instruments Ltd, U.K.). The zeta potential cell was charged with the GO dispersion with appropriate adjustments. Three zeta potential measurements were carried out for each sample, and the average is taken as the reading.

Fourier transform infrared spectroscopy (FTIR) measurements were performed for GO and GO-Ag materials (Bruker FT-IR Vertex 80). Samples were placed on top of the diamond crystal inside the ATR chamber. The special sample tightening unit that comes with the instrument was used to ensure maximum contact between the crystal and the sample. FTIR spectra were recorded from 4000 cm^−1^ to 800 cm^−1^ with a resolution of 4 cm^−1^ in the absorbance mode for 128 scans at room temperature.

Morphological characterization of the GO and GO-Ag solutions was performed using a Park Systems XE-100 AFM microscope. Imaging was carried out using a non-contact mode cantilever with a tip radius of less than 10 nm, operating at a scanning frequency of 0.5 Hz. A small droplet of GO suspension was cast onto a freshly cleaved mica piece, which was later mounted on the scanning stage of AFM after complete drying.

Micro-Raman measurements were performed with a SENTERRA (Bruker Optik GmbH) with 532 nm incident laser light and with a 10× objective. The incident laser power was adjusted accordingly to avoid sample damage and excessive heating of the sample; therefore, measurements were, performed at 2.0 mW incident laser intensity.

## Results and discussion

**Fig. 1:**
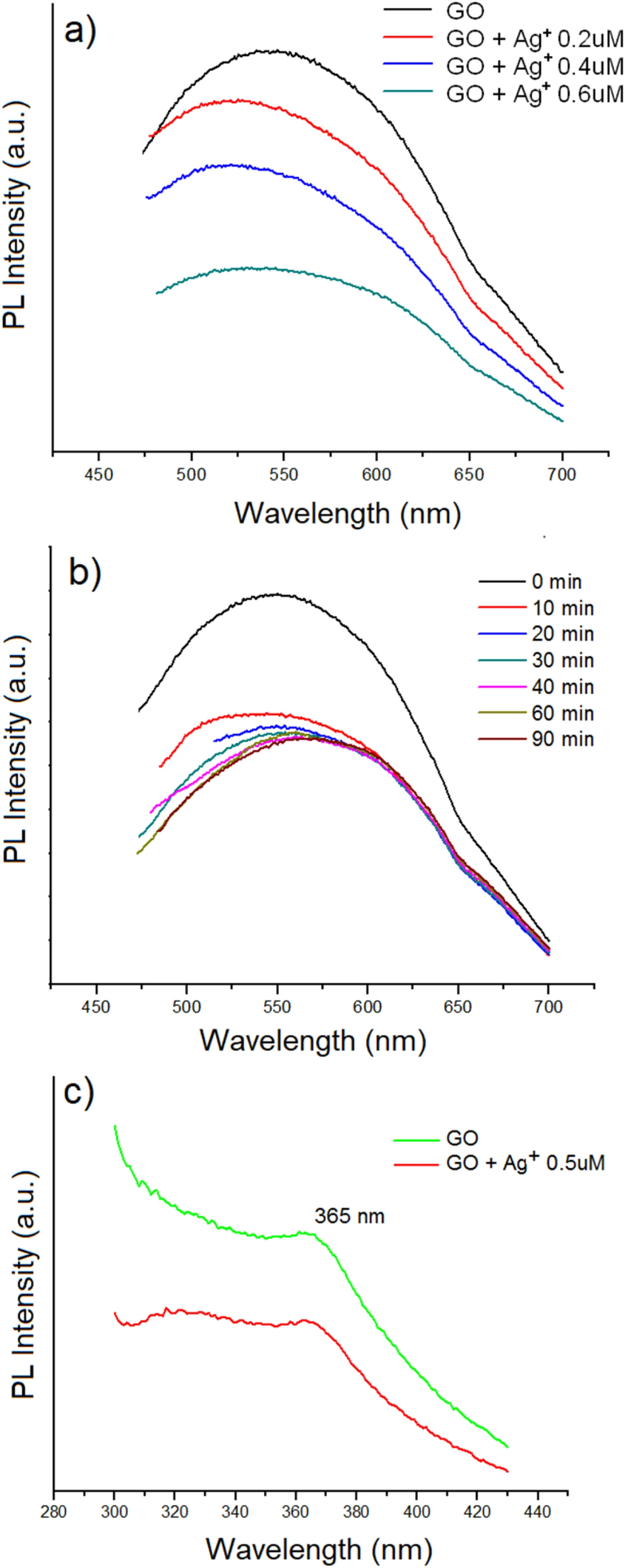
Effect of Ag^+^ ions on the PL behaviour of GO: (a) with Ag^+^ ion concentration, (b) with time, and (c) excitation spectra of both GO and GO in 0.5 µM Ag^+^ ion solution.

PL intensity of GO with varying concentrations of AgNO_3_ is shown in Fig. 1(a). Emission spectra of the exfoliated GO, after excitation at 365 nm at pH 3.5, show a broad peak centred around 530 nm, which closely follows the PL spectra described in the previous reports.^31,32,40,43^ A significant decrease in PL intensity of GO was observed with the introduction of Ag. It was also observed that, with increasing Ag^+^ concentration, PL intensity showed a decrease with no notable difference in the shape of the emission spectrum. However, a slight blue shift of the peak was observed after the addition of Ag^+^ to the GO dispersion from 530 nm to ∼520 nm in all the GO-Ag systems.

In Fig. 1(b), PL spectra of 0.5 µM AgNO_3_ and the GO system are shown with respect to time. A substantial reduction of the PL intensity was observed in the spectra even after a few minutes. A slight but continuous reduction of the PL intensity was sustained with time, which indicates that the interaction between Ag^+^ and GO is ongoing at a very slow rate. However, after 60 min, no further reduction of PL intensity was observed. PL excitation spectra of both exfoliated GO and exfoliated GO in 0.5 µM AgNO_3_ solution at 520 nm emission wavelength are shown in Fig. 1(c). Excitation features are seen in the wavelength range of 300 nm to 430 nm, with a peak excitation wavelength at 365 nm, representing the absorption energies corresponding to the emission at 520 nm. A notable reduction of the excitation spectrum was also observed for exfoliated GO in silver solution, with no significant change in the shape of the spectrum.

It was clear from the PL studies that there is a direct influence of silver addition on the PL behaviour of GO. We hypothesized that this effect is due to a specific interaction between silver ions and GO in the dispersion medium.

**Fig. 2:**
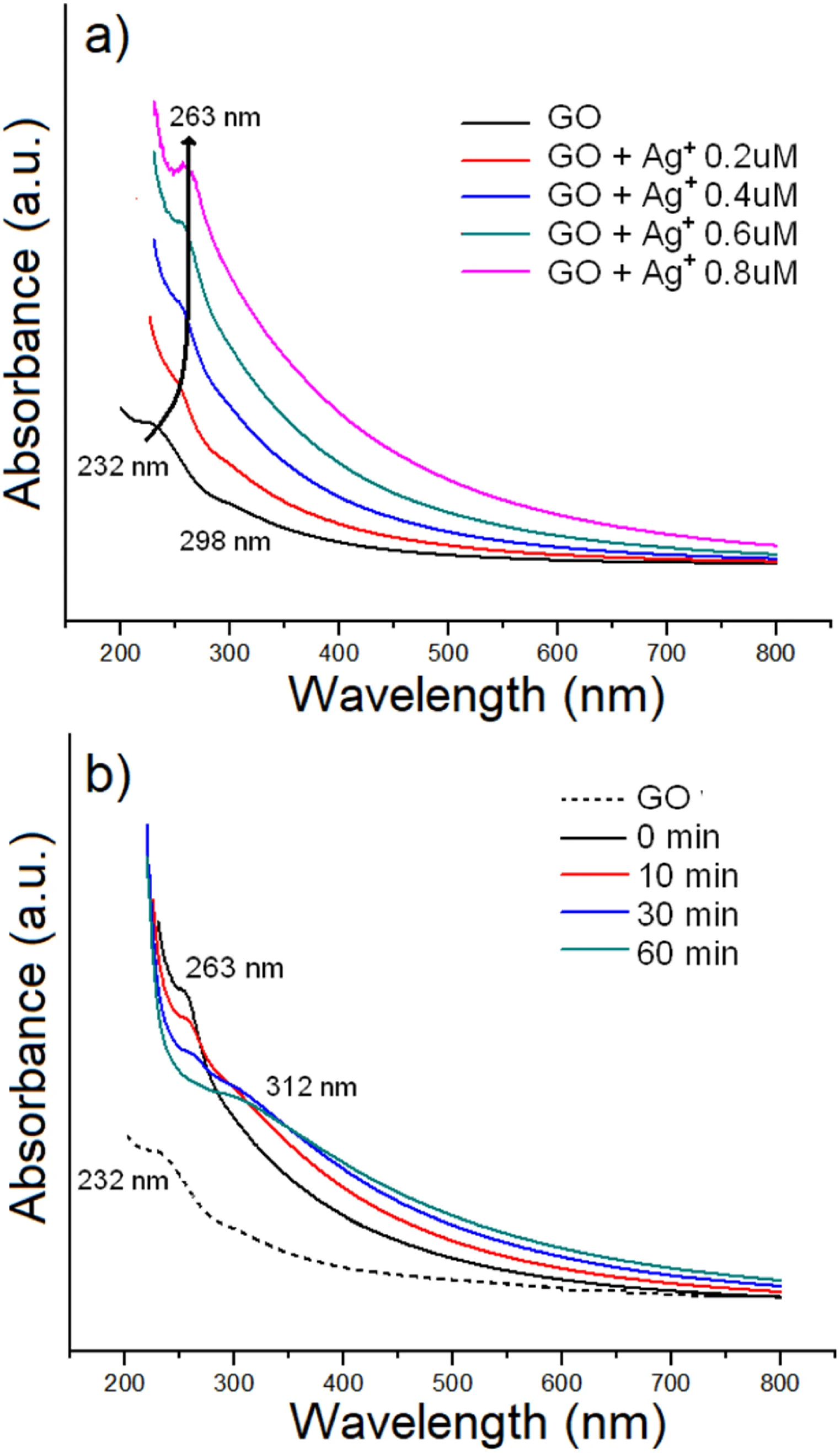
Effect of Ag^+^ ions on the absorbance behaviour of GO (a) with Ag^+^ ion concentration and (b) with time for 0.5 µM Ag^+^ ion concentration.

To further investigate the phenomenon, we carried out absorption spectroscopy. The absorption spectrum of exfoliated GO at pH 3.5 and that of the same dispersion with increasing AgNO_3_ concentrations are given in Fig. 2(a). The absorption spectrum recorded between 190 nm and 800 nm didn't show any clear peaks except the peak near 232 nm (5.4 eV) and a shoulder near 298 nm. This particular peak is consistently observed in graphitic materials such as graphene and carbon nanotubes and is known to occur due to the π-plasmon resonance.^44^ However, for carbon nanotubes, this peak tends to red shift due to their more complex band structure.^39^ The shoulder peak around 298 nm can be attributed to the n–π* transition of C=O groups.^40^ The shoulder at 298 nm disappears almost as soon as Ag^+^ is added to the solution.

On the other hand, the peak at 232 nm red shifts with the increasing concentration of Ag^+^ ions to 263 nm, while intensifying with the concentration of Ag^+^ ions introduced into the solution. A peak shift of this kind is commonly observed when GO is treated with a reducing agent to prepare reduced GO. The evaluation of oxygen, typically starting from 39% in GO to 7–8% in reduced GO, is known to increase the sp^2^ fraction of GO from 0.4 to 0.8, resulting in higher absorption in the 232 nm region.^41,45–47^

The UV-vis absorption spectra at higher Ag^+^ concentrations exhibit a pronounced tail extending into shorter wavelengths, which is primarily attributed to light scattering. This suggests that the introduction of excessive silver ions into the exfoliated GO dispersion leads to localized instability and potential agglomeration of the GO-Ag complex. It is well-documented that oxygen-bearing functional groups, specifically carboxylic, enolic, and phenolic moieties, provide the electrostatic stabilization required for aqueous GO dispersions.^46,47^ Reduction or masking of the number of these functional groups may be a reason for particle aggregation, especially in acidic media where most of the carboxylic groups are not ionized (pH 3.5).

The *in situ* reduction of Ag^+^ ions *via* galvanic displacement likely results in the masking or chemical modification of these stabilizing groups. Importantly, the fact that significant PL quenching occurs at low concentrations prior to the onset of severe scattering confirms that the quenching is fundamentally an electronic process, though it is accompanied by structural instability at higher loadings.

Therefore, we deduced that the emergence of the 232 nm peak and the evidence for strong light scattering behaviour indicate a strong chemical interaction between silver ions and GO when they are brought together in a water solution.

The absorption spectra of an exfoliated GO suspension with 0.5 µM Ag^+^ concentration recorded at different time periods are shown in Fig. 2(b). It is observed that in these spectra, peaks at 263 nm gradually decrease with time, giving birth to a much broader plasmonic peak centred around 312 nm. This interesting absorption phenomenon indicates the formation of silver nanoparticles (AgNPs) in the exfoliated graphite oxide solution.

The temporal evolution of the absorption spectra (Fig. 2(b)) reveals a significant electronic transformation. The characteristic π → π* transition of GO at 263 nm gradually diminishes, giving rise to a broad feature centered at approximately 312 nm. While larger silver nanoparticles typically exhibit a Localized Surface Plasmon Resonance (LSPR) near 400 nm, the emergence of a UV-centered feature at 312 nm is a known signature of ultra-small silver nanoclusters and nanoparticles with diameters below 5 nm. In this ultra-fine regime, the optical profile is governed by interband transitions (specifically d band to sp-conduction band transitions)^48^ and quantum size effects, which blue shift the absorption into the UV range. The broadness of the peak further suggests a polydisperse growth phase and an intimate electronic interaction between the nascent metallic clusters and the GO lattice functional groups.

**Fig. 3:**
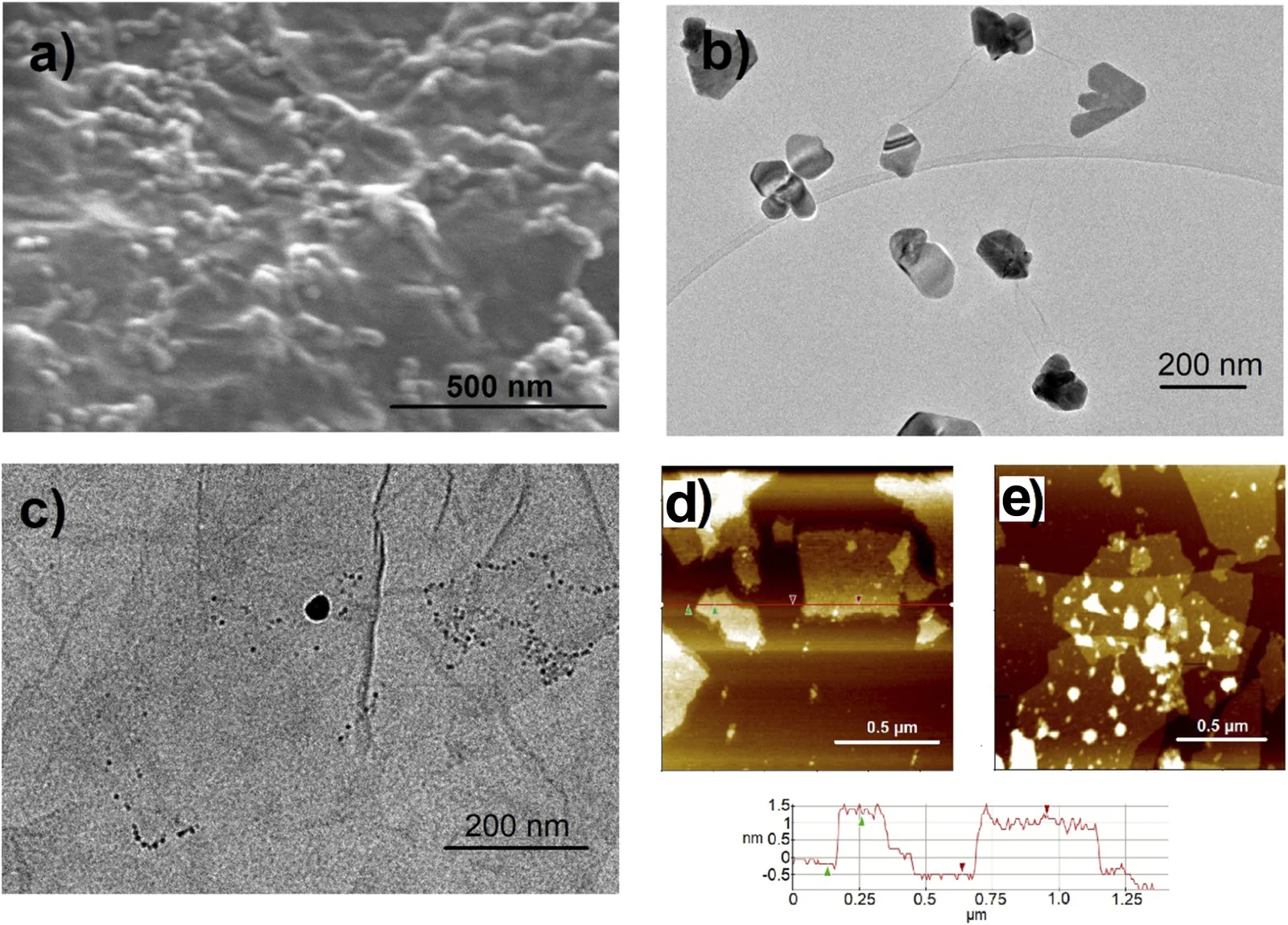
Microscopic characterization of GO/GO-Ag samples, (a) SEM image of the GO-Ag sample, (b) TEM image of AgNPs showing a frequent presence, (c) TEM image of silver nucleation points, (d) AFM image of a GO sheet, and (e) AFM image of a GO-Ag sheet showing nucleation points from GO dispersion having 0.5 µM Ag^+^ concentration.

The SEM image shown in Fig. 3a indicates a GO-Ag surface. Analysis reveals a coarse morphology with prominent wrinkles, a characteristic feature of GO.^49^ Furthermore, SEM analysis reveals the presence of particles with a spherical morphology deposited on the GO matrix. The diameter of the particles was in the range of 20–40 nm and showed a uniform distribution across the surface. These spherical particles are clearly AgNPs that result from the introduction of silver ions into the GO dispersion. The presence of AgNPs on the GO basal plane indicates that silver ions have undergone reduction after silver ions come into contact with GO in the dispersion. This observation also suggests that GO has provided a matrix for silver nucleation and growth. It's also important to note that Fig. 3(a) depicts a region with a high-density population of AgNPs. In most places, the density of the AgNPs is quite sparse, possibly owing to the very low concentration of Ag^+^ (0.8 µM) present in the GO-Ag system.

TEM was employed to analyse the morphological and structural information of the AgNPs in more detail. In the analysis, GO appeared as an almost transparent single layer GO nanosheet. Silver, being denser, appeared as dark spots in Fig. 3(b) and (c).

Analysis shows two predominant particle sizes of AgNPs in the GO matrix. The more frequent AgNP phase showed a near-spherical morphology and a scattered distribution across the sample and was seen exclusively sitting on the GO surface. Similar to SEM studies, most of the particles had diameters in the range of 20–40 nm. These results suggest that GO nanosheets play a critical role in both nucleation and growth phases of the observed AgNPs. More supportive evidence for this hypothesis can be brought forward by the presence of a minor yet prominent secondary phase of AgNPs, which had very small diameters. The diameter of these particles was smaller than 5 nm and showed to arrange themselves on the edges and individual GO sheets. The size distribution of these smaller particles was relatively narrow.

Based on these results, we suggest that, at the initial phases of the Ag^+^ and GO interaction, silver gets reduced on the edges of the GO sheets, providing nucleation points. With time, particle size would increase as they serve as nucleation centres for more silver ions to get reduced, ultimately resulting in a larger particle size. Therefore, GO nanosheets acted as a morphological seeding ground for AgNPs to nucleate and grow. The presence of two distinct AgNP phases can be explained by the very low concentration of Ag^+^ used in these experiments. Due to the very low concentration of silver ions, the growth of the particles was limited by the concentration and resulted in severe polydispersity.^50^

To further investigate the above hypothesis and to obtain more information about this smaller AgNP phase, we have carried out AFM analysis of GO platelets cast from exfoliated GO dispersion and from dispersions added with 0.5 µM AgNO_3_ after leaving for 1 hour. The results are shown in Fig. 3(e). Fig. 3(d) shows an AFM image of an exfoliated GO sheet. Complete exfoliation of GO can be confirmed by having GO plates with a thickness of 1 nm (see the insert). The surface of the GO was clear and free from any structures except random debris that may have resulted from the oxidation process (Fig. 3(b)).^51^

AFM analysis also proves that adding Ag^+^ ions to the GO dispersion clearly results in the formation of AgNP nucleation points on the surface of the GO, which complements the TEM results. According to the analysis, AgNPs with sizes ranging from 1 nm to 5 nm were seen to exclusively sit on the GO surface. Particles larger than 5 nm were not observed during the analysis.

While the present study does not provide a quantitative time-resolved particle size distribution, the collective data suggest a plausible correlation between the structural evolution of silver species and the quenching of GO fluorescence. The initial rapid decrease in PL intensity is tentatively attributed to the nucleation phase, where ultra-small clusters (<5 nm) form and create an initial electronic interface with the GO sheets. The observed saturation of quenching at ∼60 minutes likely corresponds to the transition from this nucleation regime to the growth of larger (20–40 nm) particles. It is hypothesized that, once the primary functional sites on the GO surface are modified or occupied by these initial silver nuclei, the subsequent growth into larger metallic clusters does not significantly enhance the quenching efficiency.

Several reports indicate that GO supports the spontaneous *in situ* reduction of noble metals such as gold and silver in aqueous solutions.^52–56^ Kong *et al.* used partially reduced GO to reduce HAuCl_4_ to form gold nanoparticles^57^ on the GO substrate without using any reducing agent. Xiaozhu *et al.* also reported spontaneous reduction of silver ions on both GO and reduced graphene oxide.^55^ It was reported in these studies that, when GO is brought into contact with metals such as gold or silver in the aqueous medium, spontaneous reduction of the related noble metal took place, resulting in noble metal nanoparticle-decorated GO particles. Measured particle sizes were smaller than 10 nm, which is also in agreement with the current work.

The mechanism responsible for the spontaneous reduction of Ag^+^ ions and their deposition on the GO sheets can be attributed to the galvanic displacement. This phenomenon was known to be driven by the relative potential difference between GO and silver.^58–60^ It's reported that electrons in a negatively charged material can induce the reduction of metal cations that are in contact with it,^55,57,58^ given that the reduction potentials of the donor and acceptor species are positioned feasibly for such a transfer.

The thermodynamic feasibility of this electron transfer process is determined by the relative position of the reduction potentials of both GO and silver compared to the Standard Hydrogen Electrode (SHE). If the reduction potential of GO used in our experiment is lower than that of Ag^+^, it's possible for Ag^+^ in an aqueous solution to be spontaneously reduced since the electrons can be donated from the negatively charged GO surface. This is, however, possible only if GO allows the transportation of electrons through its structure. This requirement is known to be fulfilled by GO in aqueous solutions.^61,62^

The reduction potential for silver ions at 25 °C can be obtained from the Nernst equation^63^ as given below:1
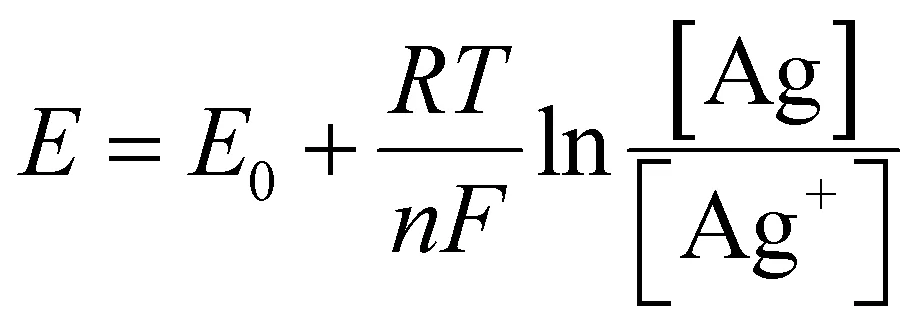
where [Ag^+^] and [Ag] are concentrations of Ag^+^ and Ag, respectively, *R* is the universal gas constant (8.314 J K^−1^mol^−1^), *T* is the absolute temperature, F is the Faraday constant (96 485C mol^−1^), and *n* is the number of electrons transferred in the half-cell reaction. *E*° (Ag^+^/Ag) + 0.80 V *vs.* SHE. When *T* = 298.15 K and [Ag^+^] is 0.5 µM, the calculated reduction potential of Ag^+^/Ag is +0.42 V.

The work function of graphene oxide is found to be ∼4.4 eV.^64^ Thus, the reduction potential of GO is −0.1 V *vs.* SHE,^65^ which is much lower than the reduction potential of Ag^+^/Ag. Therefore, it is likely that silver ions in the GO dispersion can get reduced spontaneously on the surface of the GO sheets because electrons can be donated from negatively charged GO sheets and the lower reduction potential of GO. The evidence for this electron transfer can also be seen in the XPS spectra and is discussed below.

**Fig. 4:**
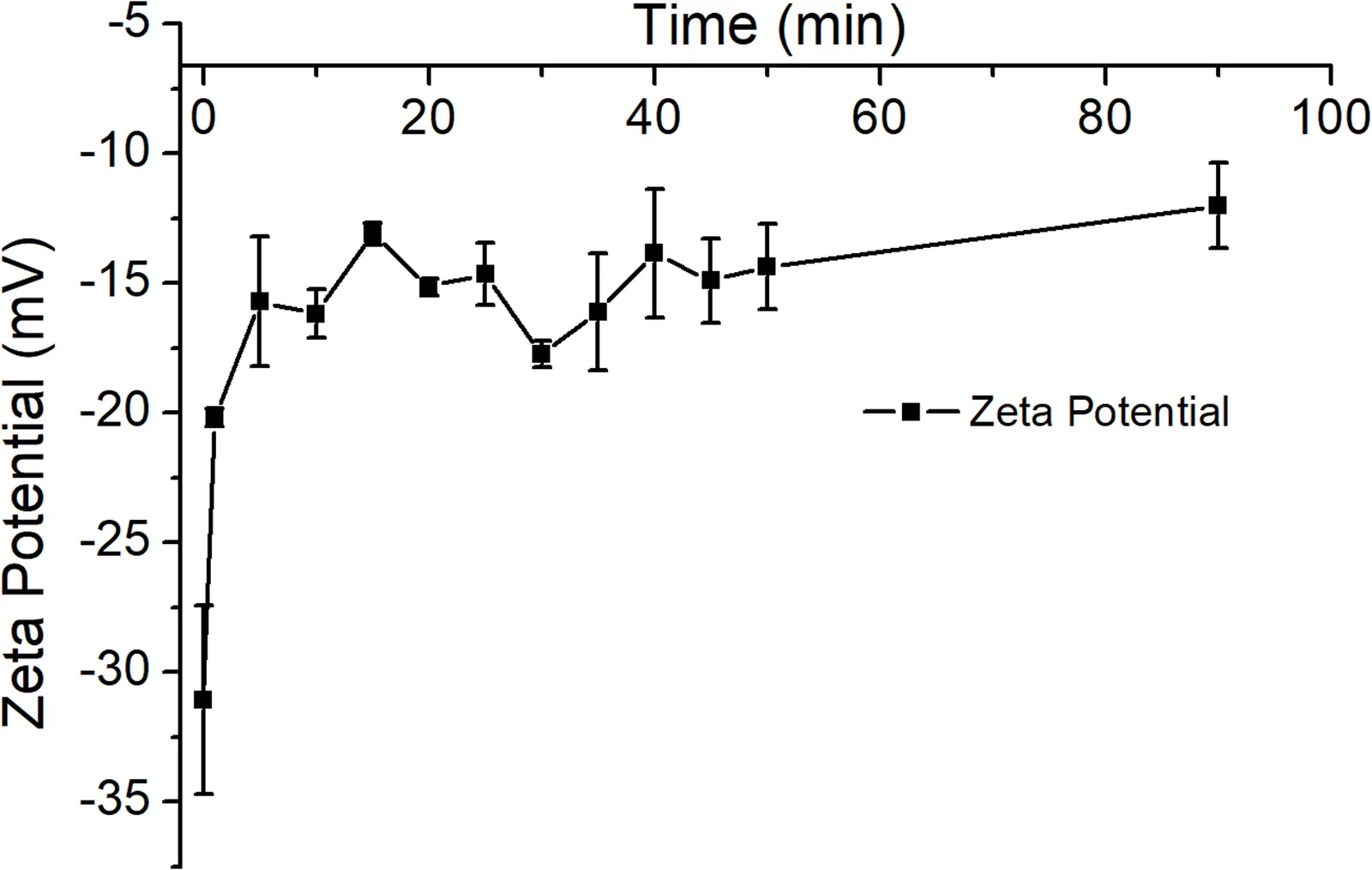
Zeta potential of aqueous GO dispersion after introduction of silver ions measured with respect to time. Note that the 0 minute measurement indicates GO before the introduction of silver ions.

To further support this hypothesis, zeta potential measurements were carried out for the GO suspension, after introducing silver ions into the solution, and the results are shown in Fig. 4.

It's seen that the zeta potential of bare GO dispersion is −31.06 ± 3.62 eV, which is consistent with previous reports.^66^ After the introduction of silver ions, a monotonic reduction in the magnitude of the zeta potential can be seen with respect to time during the initial phase. However, after ∼5 min, the zeta potential didn't show a further decrease and seemed to equilibrate around ∼15 eV. This trend and the nature of the reduction also match the PL data as well as the UV-vis data. Therefore, this net reduction of the total negative charges on GO can be considered as strong evidence for galvanic-type electron transfer from GO to silver ions.

**Fig. 5:**
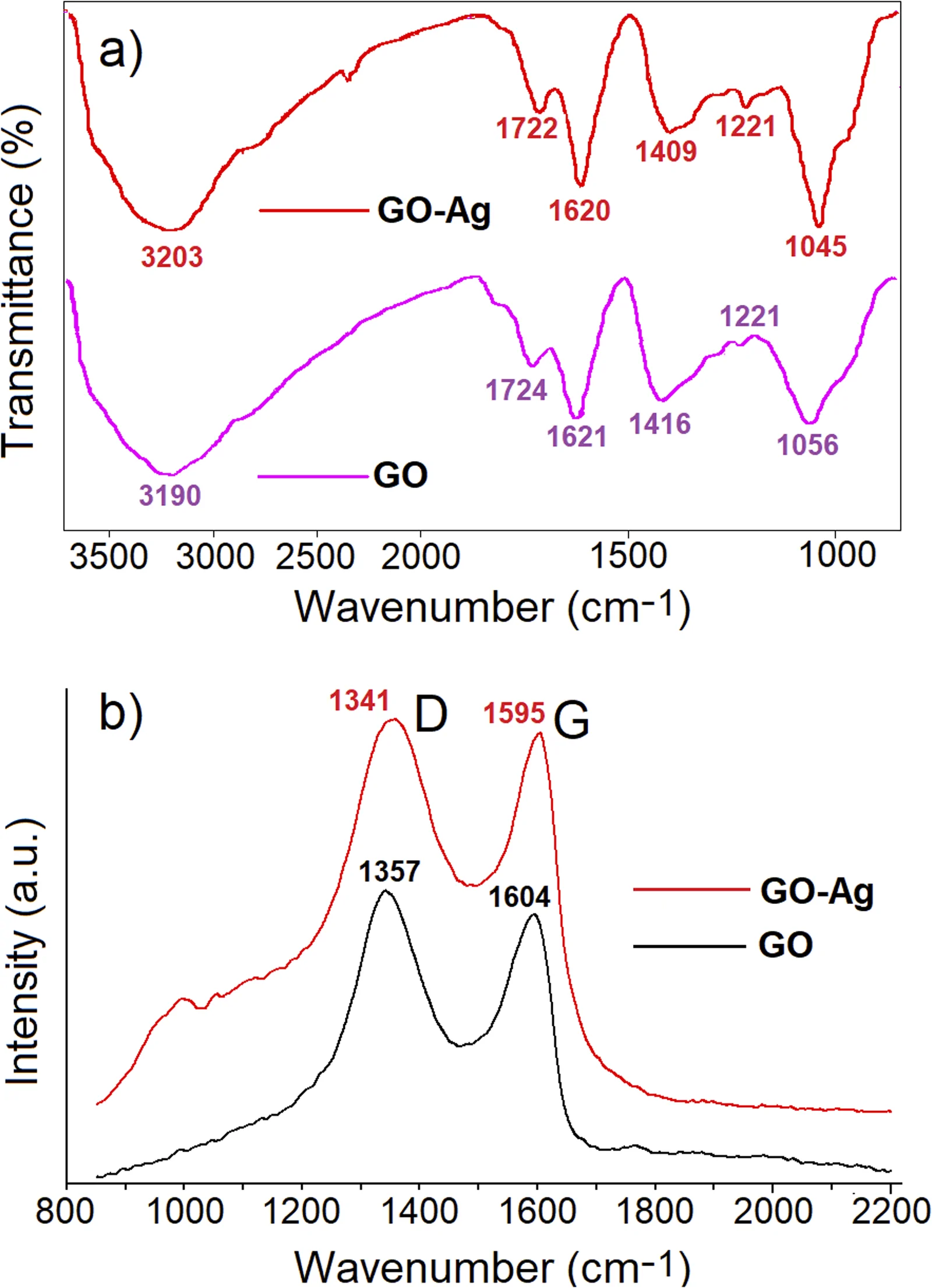
(a) FTIR spectra and (b) Raman spectra for GO and GO-Ag.

FTIR analysis was performed to investigate possible evidence of interactions between functional groups of GO and metal nanoparticles. The FTIR spectra of GO and GO-Ag are shown in Fig. 5. First, it's possible to identify several characteristic absorption bands: O–H stretching around 3203 cm^−1^ and 1409 cm^−1^, C–O stretching of the carboxylic acid carbonyl group at 1722 cm^−1^, epoxy C–O groups near 1221 cm^−1^, C–O stretching at 1045 cm^−1^, and aromatic C=C absorption at 1620 cm^−1^, which closely follow previous literature.^42^

The FTIR spectrum of GO-Ag changed little compared to that of GO in terms of shape, and no additional bands could be observed in the spectrum. However, several key changes in the band positions could be found in the GO-Ag FTIR spectrum compared to GO, which can propose possible interactions in the GO-Ag system. First, there were small blue shifts in the band from 1722 cm^−1^ in GO to 1724 cm^−1^ in GO-Ag and from 1620 cm^−1^ in GO to 1721 cm^−1^ in GO-Ag. However, these were not considered significant enough compared to the resolution of the spectrum. Second, there were significant red shifts in the bands from 1416 cm^−1^ in GO to 1409 cm^−1^ in GO-Ag and 1056 cm^−1^ in GO to 1045 cm^−1^ in GO-Ag. Both bands, 1416 cm^−1^ and 1056 cm^−1^, in GO-Ag correspond to O–H and C–O bonds in hydroxyl functionality in GO sheets, which strongly suggest that the potential interaction between GO and silver is through the hydroxyl functionality. While hydroxyl involvement is prominent, it is likely that epoxy and carboxyl groups also participate in the coordination and stabilization of the nascent silver clusters, especially given the acidic environment (pH 3.5).

Raman spectra for GO and GO-Ag are shown in Fig. 5(b). The Raman spectrum of GO is characterized primarily by two bands. The D band at 1357 cm^−1^ is assigned to the breathing of *κ*-point phonons with A_1g_ symmetry, and the G band at 1604 cm^−1^ is attributed to the tangential stretching mode of the E_2g_ phonon of the carbon sp^2^ atoms.^45^ The D band is commonly attributed to the degree of the structural imperfections created by the attachment of various oxygen-bearing functional groups, such as hydroxyl and epoxide groups, on the carbon basal plane.^45^ The G band, on the other hand, is responsible for sp^2^ hybridized carbon domains in the GO basal plane. For the spectra of GO-Ag, the peak positions of both D and G peaks shift to lower energies. Furthermore, the intensity ratio of the D band to the G band is a well-known indicator for the degree of reduction of GO. Here, the *I*_D_/*I*_G_ ratio was found to be 1.09 for GO and 1.04 for GO-Ag, which is quite insignificant.

**Fig. 6:**
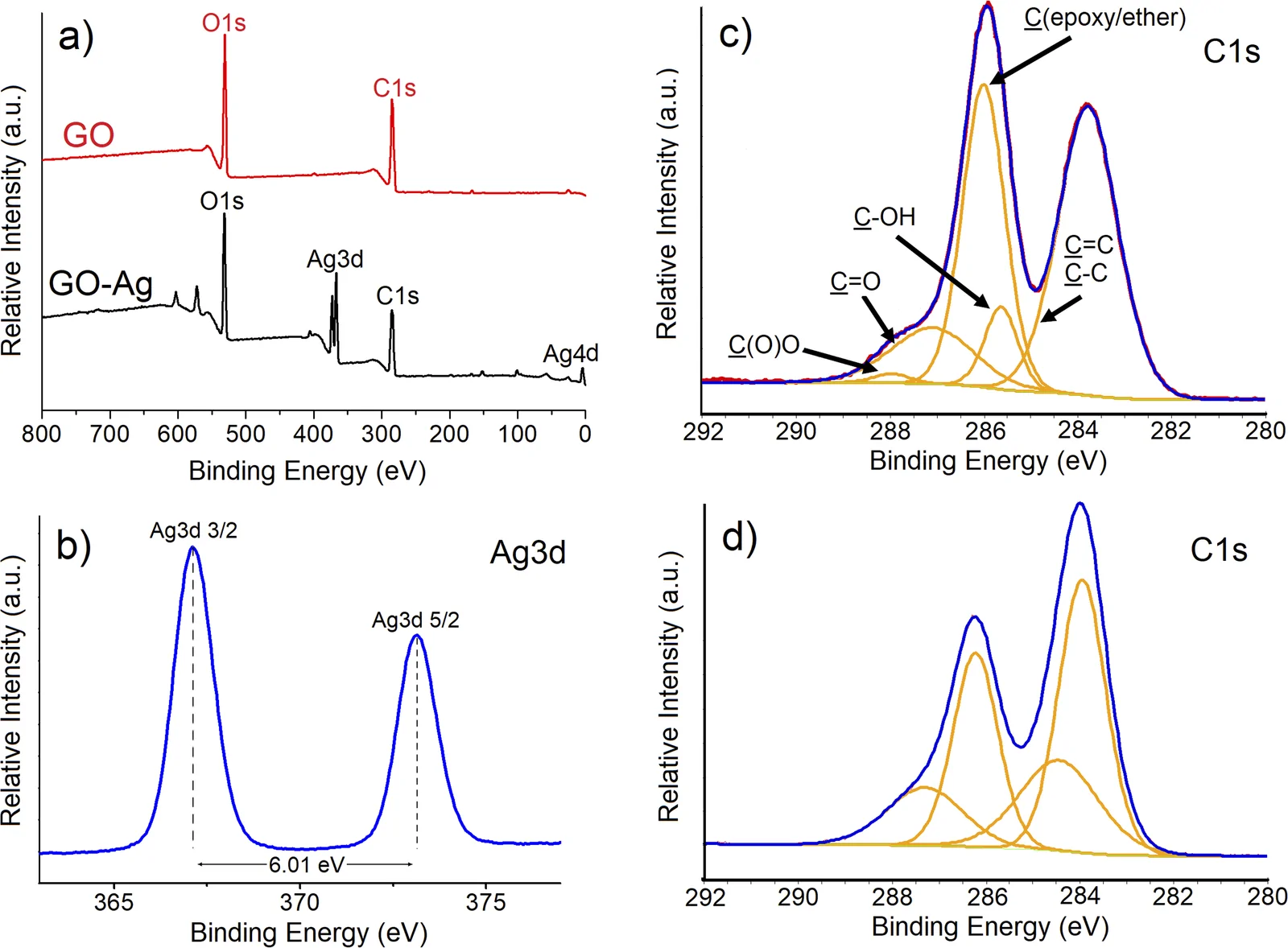
XPS spectra of (a) full survey of GO and GO-Ag, (b) high resolution spectra of the Ag 3d orbitals of GO-Ag, (c) high resolution spectra of the C 1s orbital of GO, and (d) high resolution spectra of the C 1s orbital of GO-Ag.

Fig. 6(a) shows the XPS spectra, which were obtained to identify the elements present in GO and GO-Ag structures. The peaks corresponding to binding energies (B.E.) around ∼286 eV and ∼532 eV are assigned to C 1s and O 1s orbitals, respectively. For GO-Ag samples, two additional peaks could be identified in the XPS spectrum, which were located around ∼367 eV and ∼4 eV, which were assigned to the Ag 3d orbital and Ag 4d orbital. This confirms the presence of silver in the GO matrix.

To further investigate the oxidation state and the binding nature of silver, a high resolution scan was carried out in the region related to Ag 3d B.E., as shown in Fig. 6(b). The spectrum clearly shows the signature of the Ag 3d doublet for silver, which was located at 367.1 eV and 373.1 eV. These positions are ascribed to the binding energies of Ag 3d_5/2_ and Ag 3d_3/2_ electronic states. Interestingly, these values are quite lower than the values reported for bulk metallic silver (oxidation state 0), where two bands are known to appear at ∼368.1 eV and ∼374.1 eV.^54^ However, it should be noted that the slitting of the 3d doublet of Ag was 6.0 eV, suggesting the formation of metallic silver.^67^ These shifts in Ag3d_5/2_ and Ag3d_3/2_ to lower energies than that of Ag (0) can be attributed to the charge transfer from the GO matrix to AgNPs.^52,68,69^

High-resolution spectra of the C 1s orbital are shown in Fig. 6(c). In addition to the sp^2^/sp^3^ hybridized carbon component at 283.8 eV, we've identified four band components that contribute to the overlapping of the C 1s features. Here, the component at 285.6 eV is attributed to C atoms that are directly bonded to oxygen in hydroxyl (C–OH) configurations. The component at 286.0 eV is attributed to the epoxide group (C–O–C), and two minor components at 287.1 eV and 288.0 eV are ascribed to the carbonyl groups (C=O) and carboxylic acid groups (C(O)O), respectively. These results are in close agreement with previously reported work^70,71^ and are summarized in Table 1.

**Table 1:** XPS band assignments for GO and GO-Ag

Band	GO		Band	GO-Ag	
	BE	at%		BE	at%
sp^2^/sp^3^ carbon	283.8	45.7	sp^2^/sp^3^ carbon	283.9	38.8
C–OH	285.6	7.3	C–OH	284.5	21.6
C–O–C	286.0	33.9	C–O–C	286.2	26.2
C=O	287.1	12.2	C=O/C(O)O	287.3	13.3
C(O)O	288.0	0.9			

The XPS spectrum of GO-Ag, however, was resolved into four band components. The band component at 283.9 eV is assigned to sp^2^/sp^3^-hybridized carbon, and bands located at 284.5 eV, 286.2 eV, and 287.3 eV are ascribed to C–OH, C–O–C, and C=O/C(O)O functionalities, respectively. Interesting evidence for several key changes in the GO-Ag chemical structure can be interpreted from the XPS data. First, it can be seen that, in contrast to the unmodified GO, the intensity ratio between the C–OH groups and C–O–C groups increased from 0.21 in GO to 0.82 in GO-Ag. Second, the band component position of C–OH has shifted significantly to lower energies, while the broadness of the peak increased from 0.83 eV (FWHM) to 1.99 eV (FWHM).

An increase of the C–OH/C–O–C band ratio is linked to strong interactions of metallic ions with the GO matrix across epoxide groups.^71^ This may also be partly induced by further oxidation of the GO plane due to the charge transfer effect. On the other hand, the significant red shift and the increase of the peak breadth strongly suggest possible interactions with AgNPs in proximity, which also further confirms the observations made in FT-IR analysis.

To investigate the changes induced in the valence band structure of GO after the electron transfer-driven silver nucleation and growth process, UPS analysis was carried out for both GO and GO-Ag materials. The low-energy ionizing radiation that's used in the (∼20 eV) experiment makes it possible to probe the photoelectric properties of GO, thus making it possible to investigate the valence band structure of these materials. However, it should be noted that the current experiment setup is not suitable for the exact evaluation of the work function due to changes induced by multilayer deposition^72,73^ and the insulating substrate (SiO_2_) used.

**Fig. 7:**
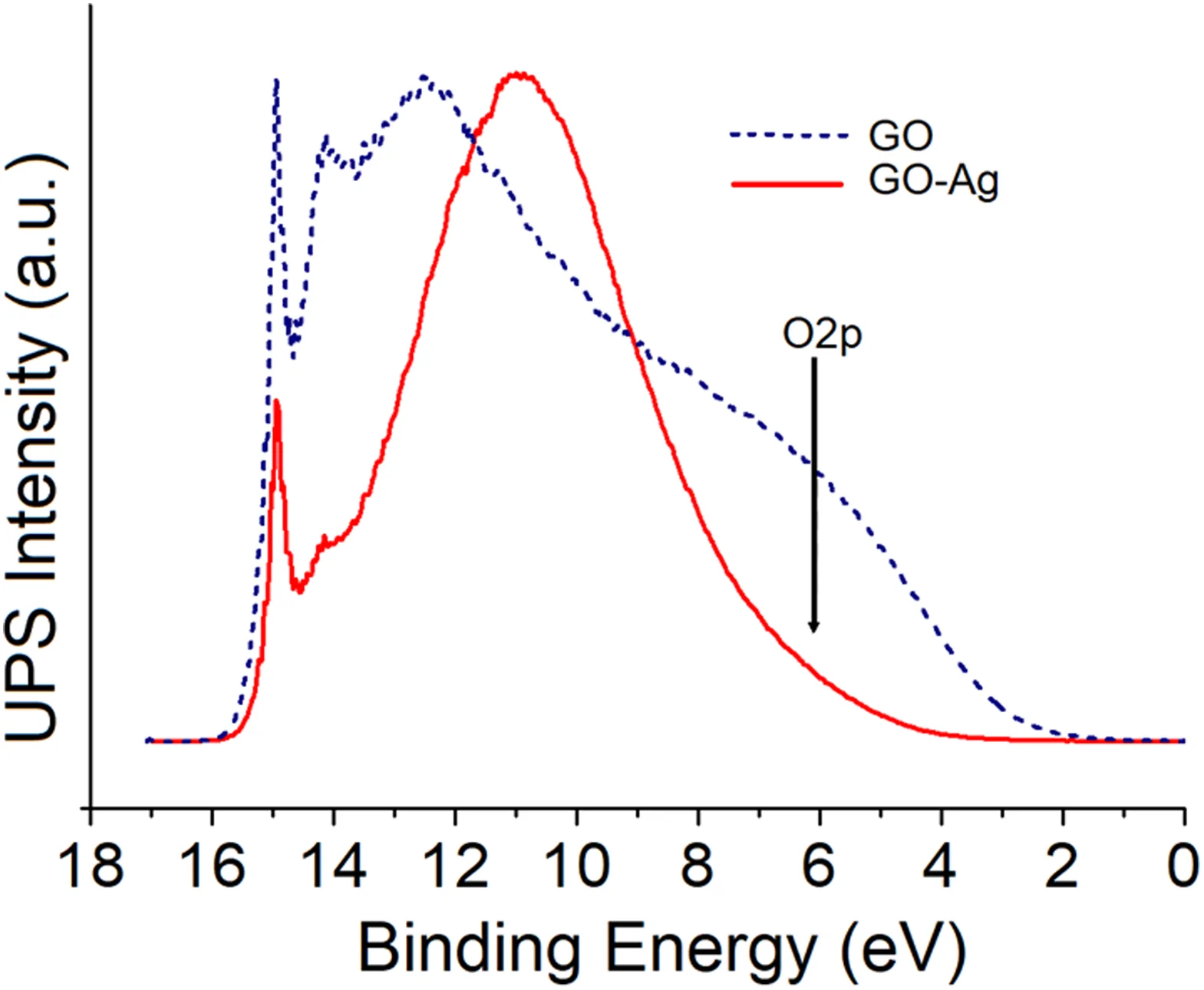
UPS spectra of GO and GO-Ag.

Valence band spectra obtained by He–I UPS measurements on GO and GO-Ag are presented in Fig. 7. As shown in the figure, the GO valence band is dominated by a broad band centred on ∼6 eV, which is assigned to O 2p states, and no states are observed at the Fermi level.^74^ Also, in the valence band region, there's no band at ∼3 eV, which is commonly observed for highly oriented pyrolytic graphite (HOPG) and reduced graphene oxide, suggesting that the hybridization of the 2pπ is poor.^74^

The high and low binding energies of HOMO cut-off positions can be determined by fitting straight lines on the spectrum graph and ruling their intersections with the binding energy axis. The Fermi level position is referred to as binding energy 0 eV. The high binding energy cut-offs were found to be 15.3 eV and 15.6 eV for GO and GO-Ag material, respectively.

The UPS spectrum of GO-Ag shows two marked differences. First, the intensity of the oxygen-related peaks, such as the O 2p peak expected at ∼6 eV, shows a significant reduction. Second, GO-Ag shows a 1.8 eV Fermi Level shift towards higher binding energies and a slight shift in the high binding energy cut-off positions, suggesting an overall increase in the material work function. Changes in the valence band structure of GO-Ag clearly suggest that interactions between the oxygen-bearing functional moieties and silver have affected the HOMO positions of GO-Ag, which explains the quenching phenomena.

The reduction of PL intensity in the GO-Ag system can be attributed to fluorescence quenching. Essentially, fluorescence quenching can be introduced as the quenching of the excited state electron singlet through a non-radiative transition back to the ground state. First, this can occur either by an energy transfer or an electron transfer process and is known to be influenced by the fluorophore type, conjugation mode, and local environment for carbonaceous compounds.^75^ Second, there can be an effective reduction of fluorophores in the matrix, which causes the fluorescence quenching.

We too hypothesize that both these reasons can contribute to the observed reduction of PL. First, PL intensity can decrease with the decrease in the effective number of fluorophores due to a chemical interaction between functional moieties contributing to the fluorescence and deposited AgNPs. Second, the PL intensity can decline due to a fluorescence quenching phenomenon driven by an energy or electron transfer mechanism that occurs between GO and AgNPs, in which the excited state energy is transferred to AgNPs through a radiative or non-radiative pathway.

Interactions between fluorophores and metallic nanoparticles are well known and extensively documented in the literature. Both fluorescent signal enhancement and quenching are observed with the presence of a nanoparticle in the vicinity. A fluorophore in its excited state behaves as an oscillating dipole. The electric field experienced by the fluorophore is affected by the incident light interaction with the metal surface.^76,77^ It's observed that with the help of experiments carried out with thin metal films, when a donor molecule is placed in the vicinity of a metal surface, not only energy transfer takes place, but also the radiative lifetimes of donor molecules also change through both radiative and non-radiative energy transfer pathways.^76–80^

The literature shows both radiative and non-radiative energy transfer between GO and other species. Quenching of certain dyes with GO is used as a method of visualizing GO sheets under a fluorescence microscope.^36^ Radiative-type fluorescence resonance energy transfer has been demonstrated with quantum dots and other species by previous research groups.^81–83^ On the other hand, non-radiative type energy transfer processes, mainly result in fluorescence quenching type interactions, are reported as well.^84^

However, we conclude that the radiative type of fluorescence resonance energy transfer (FRET) mechanism is highly unlikely in the GO-Ag system because the emission spectrum of GO (peaking at ∼530 nm) clearly doesn't overlap with the plasmonic absorption spectrum (peaking at ∼312 nm) of the AgNPs; a criterion that must be satisfied for FRET to occur.^85^

This suggests that the fluorescence quenching behaviour observed in GO-Ag systems may have gone through an electron transfer process. If this phenomenon occurs, then the interaction between GO and AgNPs deposited on the GO matrix must allow the excited state of GO fluorophores to relax non-radiatively through the interfacial charge transfer process.^86^ The efficiency of this process is attributed to the electron transfer efficiency, which is mainly determined by the band position in the excited state (lowest unoccupied molecular orbital (LUMO)) of the GO fluorophores and the work function of the AgNPs.^84^ Also, the donor and acceptor species should be in proximity, which is the case in the GO-Ag system, as observed in the microscopic analysis. It's been reported that the LUMO position of GO is −3.3 eV (ref. 87) and the work function of AgNPs is 4.7 eV. This means that the energy of the singlet state of the GO fluorophores is lower than the work function of AgNPs, rendering this quenching mechanism theoretically possible in GO-Ag structures.

However, FT-IR, XPS, and UPS data suggest another more prevailing quenching behaviour, which is due to the reduction of the fluorophore groups in the GO structure. It's reported that the origin of fluorescence in graphene oxide primarily occurs through the electronic transitions among and between the non-oxidized carbon regions and the boundary of oxidized carbon atom regions, where all types of oxygen-bearing functionalities such as C–O, C=O, and C(O)O are contributing.^31,33^ Both FT-IR and XPS provide evidence for strong interactions between AgNPs and hydroxyl functionalities in the GO matrix, a major contributor to the fluorescence behaviour of unmodified GO. Alterations made to these functional groups due to the deposition and growth of AgNPs on the GO matrix led to a reduction of the fluorescence behaviour. Furthermore, a strong reduction of O 2p states can be identified in the GO-Ag system. This presents a clear alteration to the conduction band of the fluorophore, leading to the observed behaviour.

So we conclude that the quenching of the PL intensity of GO can be attributed to two distinct mechanisms. First, the deposition and growth of AgNPs on the GO matrix induce a chemical change in functional groups attached to the basal plane, which in turn reduces the PL intensity. Second, there can be a feasible electron transfer from the excited fluorophore of GO to AgNPs due to the relative band positions and work function of silver.

## Conclusions

PL behaviour of GO prepared with the improved Hummers' method was observed. The addition of Ag^+^ ions to the exfoliated GO solution led to the formation of small nanoparticles on the GO plates. Significant quenching of PL yield was observed after the addition of Ag^+^ ions. Reduction in the PL yield of GO after adding Ag^+^ to the solution was found to be linked to the rapid formation of silver nucleation points, followed by the growth of the particles to larger sizes. The reduction of silver ions to silver (0) was attributed to the galvanic displacement of electrons from the GO matrix to silver ions and particles. Both XPS and FT-IR suggested that the main silver–GO interaction moiety is the hydroxyl group. We concluded that fluorescence quenching behaviour is due to both alteration of the oxygen-bearing functional moieties on the GO basal plane and electron transfer from the excited GO fluorophore to AgNPs, relaxing the excited state electron singlet through non-radiative type relaxation back to the ground level.

The comprehensive characterization of the GO-Ag interface presented here directly informs several potential applications. Potential for the modulation of the work function and the preservation of the carbon sp^2^ lattice are critical for the development of high-performance photocatalysts and transparent conductive electrodes. Specifically, the electronic coupling at the interface provides the physical basis for enhanced charge carrier separation, which is the primary driver for efficiency in solar energy conversion and sensing devices. Thus, while this work focuses on fundamental interfacial properties, the findings serve as a necessary design protocol for integrating these composites into functional electronic and catalytic systems.

## Conflicts of interest

There are no conflicts to declare.

## Data Availability

The data supporting this article are available within the manuscript and in an external online repository. This includes: • Primary characterization data: detailed fluorescence (PL) quenching spectra (Fig. 1), UV-visible absorption and π-plasmon resonance analysis (Fig. 2), and electron microscopy (SEM and TEM) and AFM morphological studies (Fig. 3) are included within the article. • Surface and electronic analysis: zeta potential measurements (Fig. 4), FTIR and Raman spectroscopic data (Fig. 5), and chemical state analysis *via* XPS (Fig. 6 and Table 1) and UPS valence band structure mapping (Fig. 7) are contained in the main text. • Raw numerical datasets: supporting raw data files, including numerical records for pH-dependent fluorescence and concentration-dependent photoluminescence intensity, are openly accessible at the following link: https://drive.google.com/drive/folders/1pQQMWnzFkGdljXNOmTxA_LV63iiTjWBK?usp=drive_link.
